# A new hereditary colorectal cancer network in the Middle East and eastern mediterranean countries to improve care for high-risk families

**DOI:** 10.1007/s10689-017-0018-6

**Published:** 2017-07-06

**Authors:** Zeinab Ghorbanoghli, Carol Jabari, Walid Sweidan, Wail Hammoudeh, George Cortas, Ala I. Sharara, Amal Abedrabbo, Ijad Hourani, Bahareh Mahjoubi, Keivan Majidzadeh, Nurdan Tözün, Hadia Ziada-Bouchaar, Waseem Hamoudi, Osama Diab, Hamid Reza Khorram Khorshid, Henry Lynch, Hans Vasen

**Affiliations:** 1 Department of Gastroenterology and Hepatology, Leiden University Medical Centre & Netherlands Foundation for the Detection of Hereditary Tumours, Leiden, The Netherlands; 2 Patient’s Friends Society, Jerusalem, Palestine; 3 Department of Gastroenterology, Makased Islamic Charitable Hospital, Jerusalem, Palestine; 4 Department of Internal Medicine, Arabcare Hospital, Ramallah, Palestine; 5 Department of Gastroenterology, St. George Hospital Medical Center, University of Balamand Medical School, Beirut, Lebanon; 6 Division of Gastroenterology, American University of Beirut Medical Center, Beirut, Lebanon; 7 Department of Pediatrics, Makased Islamic Charitable Hospital, Jerusalem, Palestine; 8 Department of Surgery, Agusta Victoria Hospital, Jerusalem, Palestine; 9 Colorectal Research Center, Iran University of Medical Sciences, Tehran, Iran; 10 Motamed Cancer Institute, ACECR, Tehran, Iran; 11 Department of Internal Medicine and Gastroenterology, University of Acibadem, Acibadem Kozyatagi Hospital, Istanbul, Turkey; 12 Laboratory of Biology and Molecular Genetics, Faculty of Medicine, University 3, Constantine, Algeria; 13 Department of Gastroenterology, The Royal Hospital, Amman, Jordan; 14 Department of Internal Medicine, Creighton University, Omaha, USA; 15 Genetic Research Center, University of Social Welfare and Rehabilitation Sciences, Tehran, Iran; 16 Creighton’s Hereditary Cancer Center, Creighton University, Omaha, USA; 17 Department of Gastroenterology & Hepatology, Leiden University Medical Centre, P.O. Box 9600, 2300 RC Leiden, The Netherlands; 18 Netherlands Foundation for the Detection of Hereditary Tumours, Leiden, The Netherlands; 19 Hebron University, Hebron, Palestine

**Keywords:** Lynch syndrome, Familial colorectal cancer, CMMRD, Health care, Identification, Registry

## Abstract

Colorectal cancer (CRC) has a very high incidence in the western world. Data from registries in the Middle East showed that the incidence of CRC is relatively low in these countries. However, these data also showed that CRC incidence has increased substantially over the past three decades and that a high proportion of cases are diagnosed at an early age (<50 years). In view of these findings, more attention should be paid to prevention. Because of the often limited financial resources, focused screening of individuals with hereditary CRC, in particular those with Lynch syndrome, appears to be the most cost-effective strategy. During recent meetings of the Palestinian Society of Gastroenterology and the Mediterranean Task force for Cancer Control (MTCC) in Jericho, and the Patient’s Friends Society of Jerusalem in Hebron the issue of hereditary CRC in the Middle East was discussed and the idea was conceived to establish a network on hereditary colorectal cancer (HCCN-ME) with the goal of improving care for high-risk groups in the Middle East and (Eastern) Mediterranean Countries.

## Introduction

In the western world, colorectal cancer (CRC) has a very high incidence, occupying the second or third position on the list of commonest cancers in most countries (http://Globocan.iarc.fr). Detailed information on the epidemiology of CRC in the Middle East is limited to the few cancer registries currently in existence. Data from the Gharbia registry (Egyptian region with 4 million inhabitants) showed that the incidence of CRC is relatively low (5/100,000), with higher rates in urban areas compared to rural areas [1]. Data from the Israel National Cancer Registry confirms the low incidence of CRC in the Arab population (Israel National Cancer Registry website). However, data from this registry also showed that CRC incidence has increased substantially over the past three decades, with the age standardized rate (ASR) increasing from 5.5/100,000 in the 1980s to around 20/100,000 in 2013.

An interesting characteristic of the epidemiology of CRC in the Middle East is the high proportion of cases that are diagnosed at an early age. For example, 24% of the CRC cases in the Gharbia district in Egypt and 25% of CRC cases in Iran were diagnosed before ages 40 and 45, respectively [1, 2]. These observations may be partly explained by the different age structure of the population, with a higher proportion of the population age <50 in the Middle East compared to the Western countries.

In view of the increasing incidence of CRC and the early onset of CRC in Middle East countries, greater attention should be paid to the prevention of CRC in this region.

## Prevention of CRC

In general, two options for prevention of CRC are available: (1) offering screening to the general population using fecal occult blood testing (FOBT), or (2) focusing screening on high risk groups. The first option has already been implemented in many Western countries. Experience with a nationwide FOBT screening program (age: 55–75) that started in the Netherlands in 2014 is very promising. The participation rate is very high (>70% out of 4 million people) and the yield of colonoscopies is substantial, with 8% CRC and >40% advanced adenomas (http://www.rivm.nl). However, a large-scale population-based program is relatively expensive (>80 million Euro’s per year in the Netherlands). In view of the lower incidence of CRC in the Middle East and limited financial resources, focusing screening on high-risk groups might be more cost-effective.

## What proportion of CRC in the Middle East is familial or hereditary?

**Fig. 1:**
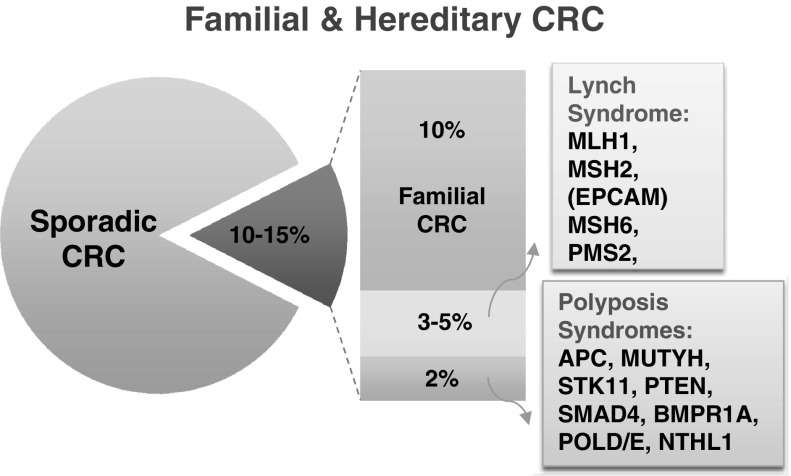
Etiology of colorectal cancer (CRC)

Studies in Western countries have shown that 10–15% of all CRCs are caused by hereditary factors (Fig. 1). About 10% is caused by clustering of CRC in families without an identified high penetrance gene defect, a group referred to as familial colorectal cancer. Approximately 2% is associated with polyposis syndromes, mainly the syndromes caused by mutation(s) in the *APC* gene (familial adenomatous polyposis) and *MUTYH* (*MUTYH*-associated polyposis) [3].

The most common hereditary CRC syndrome is Lynch syndrome, which is responsible for 3–5% of all CRC cases. This autosomal dominant syndrome is caused by mutations in one of the mismatch repair (MMR) genes (*MLH1*, *MSH2* (*EPCAM*), *MSH6* and *PMS2*). The main features of the syndrome are (1) an early age of onset of CRC, (2) a high risk of developing multiple tumors, and (3) an association with the development of other cancers (endometrium, ovaries, urinary tract, stomach and other sites). The hallmark of the syndrome is the presence of microsatellite instability (MSI) in the tumor. Several studies from the Middle East reported Lynch syndrome families, and the clinical features of Lynch syndrome families appear to be similar to those seen in Western families [4].

A study from Egypt, focused on obtaining a detailed family history in a series of consecutive patients with CRC, suggested that 7–14% of cases show evidence for familial or hereditary CRC [5]. Other studies evaluated the presence of MMR deficiency in various countries in the Middle East with CRC and found evidence for MMR deficiency in approximately 11–20% of tumors [6–8]. These data thus suggest that the proportion of familial or hereditary CRC in the Middle East is probably comparable to that in Western countries.

## How can Lynch syndrome families be identified in the Middle East?

Obtaining a detailed family history is the most cost-effective approach to identifying familial and hereditary CRC. If facilities for tumor analysis [MSI or immunohistochemical analysis of the MMR-proteins (IHC)] are limited, the classic clinical criteria (revised Amsterdam criteria: 3 CRCs or associated cancers in two successive generations, one cancer diagnosed before age 50 years) [9] can be used to assess whether one is dealing with a Lynch syndrome family. However, families that comply with only some of these criteria should not be excluded from colonoscopic surveillance.

Currently, the best way to identify Lynch syndrome families is by applying MSI analysis or immunohistochemical analysis (IHC) of tumor MMR protein expression. This analysis of CRC tumors can be performed in suspected families based on the main features of Lynch syndrome: early age of onset (<50 years), the occurrence of multiple tumors or clustering of CRC in the family. A relatively new approach is systematic screening by MSI or IHC of all newly diagnosed CRC <70 years, a method that is now being implemented in many Western countries. In addition, an increasing numbers of studies are reporting the testing of multiple cancer-associated genes by next generation sequencing (NGS) [10, 11].

## The importance of recognizing CMMRD in the Middle East

A distinct means to identify Lynch syndrome families in the Middle East is through recognition of constitutional mismatch repair deficiency (CMMRD) [12, 13]. This is a very rare autosomal recessive syndrome characterized by the development of multiple tumors in the first and second decade of life. These malignancies include brain tumors, cancer of the colon and small bowel, and hematological malignancies such as leukemia and non-Hodgkin lymphoma. Other features include the development of multiple colorectal adenomas in the second decade of life and the presence of café au lait spots. CMMRD is caused by two germline mutations in the same MMR gene. Marriage between cousins, especially in rural areas, is relatively common in the Middle East and this custom is therefore also likely in families with Lynch syndrome. Parents who are both affected with or predisposed to Lynch syndrome have a 25% risk of having a child who develops CMMRD.

Lynch syndrome should therefore be considered when a child has features of CMMRD (Table 1). However, as the syndrome is typically caused by mutations in *PMS2*, a gene associated with a relatively low CRC penetrance, CRC might even be absent in family members of a CMMRD patient. CMMRD diagnosis can be made by immunohistochemical analysis of the MMR proteins in both normal and tumor tissue, and by germline mutation analysis.

**Table 1:** Features of constitutional mismatch repair deficiency (CMMRD) [13]

Feature	Age range at diagnosis (median)
*Hematological malignancies*	
Non-hodgkin lymphoma	0.4–17 (5 years)
Lymphoid leukemia	2–21 (6 years)
Acute myeloid leukemia	6–17 (9.5 years)
*Malignant brain and central nervous system tumors*	
High-grade gliomas	2–40 (9.5 years)
sPNET	4–17 (8 years)
Medulloblastoma	4–12 (7 years)
*LS-associated carcinomas*	
Colon/rectum	8–48 (16 years)
Duodenum/jejunum/ileum	11–42 (28 years)
Endometrium	23–44 (28 years)
Bladder/ureter/renal pelvis/ovaries	unknown
*Benign features*	
Adenomas/polyps of colon, rectum and duodenum	6–46 (14 years)
Café au Lait	

## Middle East network for hereditary CRC

In conclusion, in view of the increasing incidence of CRC in the Middle East and the substantial number of early onset CRC cases, more attention should be paid to prevention. In light of often limited financial resources, focused screening of individuals with hereditary CRC, in particular those with Lynch syndrome, appears to be the most cost-effective strategy.

During recent meetings of the Palestinian Society of Gastroenterology and the Mediterranean Task force for Cancer Control (MTCC) in Jericho, and the Patient’s Friends Society of Jerusalem in Hebron on April 7 and 8, 2017, the issue of hereditary CRC in the Middle East was discussed and the idea was conceived to establish a* Middle East Network on Hereditary Colorectal Cancer (HCCN-ME)*. The main goal of this new Network is to improve care for these high-risk groups in the Middle East and Eastern Mediterranean Countries. Our activities will include (1) an inventory of current care in the countries concerned, (2) development of guidelines for diagnosis and management, (3) development of patient information pamphlets, (4) identification of referral centers for genetic counseling, MSI analysis and DNA analysis in the various countries/regions, (5) establishing registries to guarantee lifetime surveillance, and (6) development of a website. Individuals and organizations in Middle East countries and other (Eastern) Mediterranean countries interested in participating in this network are invited to contact the Secretariat of the network: Dutch Hereditary Colorectal Cancer Registry; Leiden, The Netherlands; hfavasen@stoet.nl. Information on the network can be found at: http://www.hccn-me.com.
